# Integrating Multi-omics Data to Dissect Mechanisms of DNA repair Dysregulation in Breast Cancer

**DOI:** 10.1038/srep34000

**Published:** 2016-09-26

**Authors:** Chao Liu, Florian Rohart, Peter T. Simpson, Kum Kum Khanna, Mark A. Ragan, Kim-Anh Lê Cao

**Affiliations:** 1 Institute for Molecular Bioscience, The University of Queensland, St. Lucia, QLD 4067, Australia; 2 The University of Queensland Diamantina Institute, The University of Queensland, Woolloongabba, QLD 4102, Australia; 3 UQ Centre for Clinical Research and School of Medicine, The University of Queensland, Herston, QLD 4101, Australia; 4 QIMR-Berghofer Medical Research Institute, Herston, Brisbane, QLD 4006, Australia

## Abstract

DNA repair genes and pathways that are transcriptionally dysregulated in cancer provide the first line of evidence for the altered DNA repair status in tumours, and hence have been explored intensively as a source for biomarker discovery. The molecular mechanisms underlying DNA repair dysregulation, however, have not been systematically investigated in any cancer type. In this study, we performed a statistical analysis to dissect the roles of DNA copy number alteration (CNA), DNA methylation (DM) at gene promoter regions and the expression changes of transcription factors (TFs) in the differential expression of individual DNA repair genes in normal versus tumour breast samples. These gene-level results were summarised at pathway level to assess whether different DNA repair pathways are affected in distinct manners. Our results suggest that CNA and expression changes of TFs are major causes of DNA repair dysregulation in breast cancer, and that a subset of the identified TFs may exert global impacts on the dysregulation of multiple repair pathways. Our work hence provides novel insights into DNA repair dysregulation in breast cancer. These insights improve our understanding of the molecular basis of the DNA repair biomarkers identified thus far, and have potential to inform future biomarker discovery.

Cells have evolved complex mechanisms to repair DNA lesions that arise from various endogenous and exogenous factors, including ultraviolet radiation, chemical carcinogens and oxidative by-products from normal cellular respiration. Hundreds of DNA repair genes have been identified, which mainly participate in five distinct but functionally intermingled pathways: homologous recombination (HR), non-homologous end joining (NHEJ), nucleotide excision repair (NER), base excision repair (BER) and mismatch repair (MMR). The functionalities of these pathways and their constituent components have been elucidated in detail^1^,^2^,^3^.

DNA repair genes and pathways that are transcriptionally dysregulated in tumours carry valuable information with regard to drug response, patient survival and tumour characteristics, and thus have been extensively studied for biomarker discovery^4^,^5^,^6^,^7^,^8^,^9^,^10^. For instance, Santarpia *et al.*^8^ analysed the expression profiles of 145 DNA repair genes in untreated breast cancer patients versus breast cancer patients treated with chemotherapeutic agents. The authors found that the upregulation of nine genes (*BUB1*, *FANCI*, *MNAT1*, *PARP2*, *PCNA*, *POLQ*, *RPA3*, *TOP2A*, and *UBE2V2*) are associated with poor prognosis, and that of one gene (*ATM*) is associated with good prognosis^8^. At the pathway level, Kang *et al.*^7^ devised a DNA repair pathway-focused score (DRPFS) by combining the expression levels of 23 genes involved in platinum-induced DNA damage repair; this DRPFS score outperforms other clinical factors in predicting treatment response of ovarian cancer patients^7^. More recently, our group^9^ developed an HR score based on the expression of about 70 core HR genes in breast cancer. This score reflects HR repair efficiency and correlates with chromosomal instability as well as breast cancer patient survival^9^. While the dysregulation of DNA repair genes and pathways has been documented in many studies, to our knowledge, the molecular mechanisms underlying these transcriptional abnormalities have not been systematically elucidated in any cancer type.

Cancer-related gene expression alterations may result from genetic and/or epigenetic changes in tumours, including DNA copy number alteration (CNA) and DNA methylation (DM) of CpG islands at gene promoter regions. Interestingly, aberrantly expressed genes with CNA or DM are good candidates for cancer driver genes. For example, *MYC* was considered an oncogene candidate as its overexpression together with its copy-number gain were commonly observed in cancer^11^, leading to subsequent experiments that further validated its oncogenic role^12^. The recent availability of multi-omics data in several major cancers has facilitated a more-holistic understanding of the global impact of CNA or DM on the transcriptomic changes^13^,^14^,^15^. However, effects specific to DNA repair dysregulation have yet to be elucidated.

Transcription factors (TFs) are key cellular components that serve to activate or repress the transcription of their target genes. Cancer-related expression changes of TF genes are often crucial events as they are frequently associated with tumour initiation and/or development. For example, a recent meta-analysis revealed that the transcriptional regulatory network in colorectal adenomas is characterised by more than 250 differentially expressed TF genes, a considerable fraction of which have established roles in colorectal tumourigenesis^16^.

Identifying target genes for individual TFs is challenging. Motif-based computational prediction of TF binding sites at gene promoter regions has long been used to infer TF-target relationships^17^,^18^; however, it is a well-known issue that such analyses tend to give false positive results mainly due to the short length of the motifs and a lack of tissue specificity. In recent years, ChIP-Seq, which combines chromatin immunoprecipitation (ChIP) with massively parallel DNA sequencing, has been employed to produce genome-wide TF binding profiles in a cell line-specific manner. This technique can generate relatively accurate information regarding TF binding sites; however, due to its high cost, as of now only a limited number of TFs have been profiled in certain cell lines^19^. Moreover, for TFs whose binding profiles have been measured by ChIP-Seq, defining their target genes still remains an open question^20^.

Breast cancer is one of the most common malignancies worldwide. This malignancy has a particularly close relationship with DNA repair defects, with the two well-known breast cancer susceptibility genes, *BRCA1* and *BRCA2*, being essential components of the HR repair pathway^21^,^22^. Previous studies showed that DNA repair genes and/or pathways are frequently dysregulated in breast cancer^8^,^9^,^10^. In this study, we aimed to provide biological insights into the underlying mechanisms of DNA repair dysregulation in this cancer type. Taking advantage of the breast cancer multi-omics data recently generated by the Cancer Genome Atlas (TCGA)^15^, we first identified DNA repair genes that are differentially expressed between normal and tumour samples. Next, we evaluated the in *cis* effects of CNA and DM on the expression alterations of the identified repair genes. Finally, we developed a penalised linear regression-based statistical framework, which takes into account the effects of CNA and DM on gene expression, to select TFs potentially associated with each differentially expressed DNA repair gene. Our results showed that CNA and the transcriptional changes of the identified TF can statistically explain most of the expression variance of the repair genes, indicating the potential importance of these two factors in driving DNA repair dysregulation in breast cancer.

## Results

### Identification of DNA repair genes that are differentially expressed between tumour and normal breast tissues

Our analysis is based on 195 DNA repair genes that we manually curated (Supplementary Table S1; published in part in ref. 3). These genes participate in the five major DNA repair pathways and the Fanconi anaemia (FA) pathway, which is responsible for the repair of DNA inter-strand crosslinks and is closely associated with breast cancer susceptibility^23^. Of these 195 repair genes, 169 have CNA, DM and expression data in TCGA, of which 149 (88%) are differentially expressed between normal and tumour breast samples (Table 1; Supplementary Table S2). This high percentage of differential expression is consistent with the existing knowledge that DNA repair genes are frequently dysregulated in breast cancer. Of the 149 differentially expressed repair genes, 106 (71%) exhibit significantly increased expression, and 43 (29%) show reduced expression. Similar observations were obtained when the numbers of up- and down-regulated genes within each individual repair pathway were examined separately (Table 1), indicating that DNA repair genes are more likely to be up-regulated than down-regulated in breast cancer.

### Estimation of the effects of genetic and epigenetic changes on the DNA repair dysregulation in breast cancer

#### Contribution of CNA to the DNA repair dysregulation

**Figure 1:**
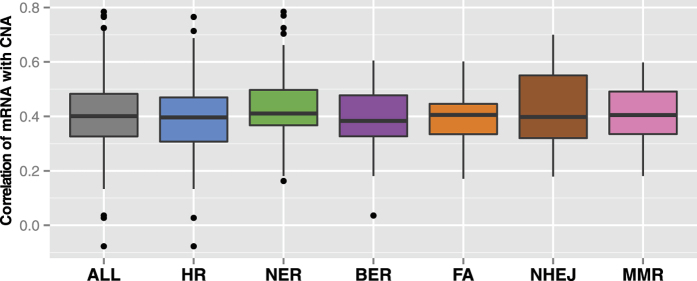
The effects of CNA on DNA repair gene expression. Distributions of the in *cis* Spearman correlations between CNA and mRNA expression, summarised for all differentially expressed DNA repair genes, or only genes from each repair pathway.

**Figure 2:**
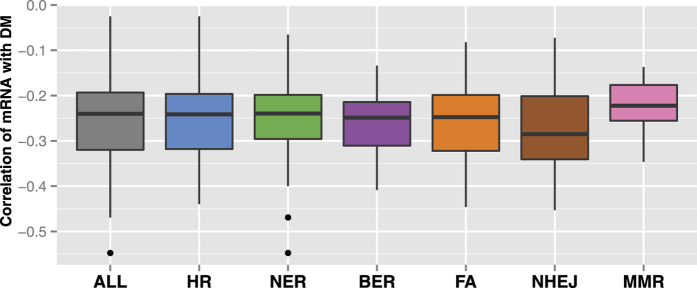
The effects of DM on DNA repair gene expression. Distributions of the in *cis* Spearman correlations between DM and mRNA expression, summarised for all differentially expressed DNA repair genes, or only genes from each repair pathway.

To evaluate the effects of CNA and DM on DNA repair dysregulation, for each of the differentially expressed repair genes (Table 1), we measured the respective correlations of mRNA with CNA (Fig. 1 and Table 2) and DM (Fig. 2 and Table 3) using Spearman correlation coefficients.

As shown in Fig. 1, the correlations between CNA and mRNA are in general modest, with a median correlation coefficient of about 0.4 (Supplementary Table S3). Out of the 149 differentially expressed repair genes, 148 show positive correlations between CNA and mRNA, of which 146 have significant correlations (FDR < 0.05; Supplementary Table S3). These positive correlations are consistent with the role of CNA in modulating gene expression, and the modest values indicate that CNA plays a nontrivial role in driving DNA repair dysregulation in breast cancer. Similar patterns were observed when either all differentially expressed repair genes were considered, or when only genes within each repair pathway were included (Fig. 1), indicating that CNA affects different repair pathways in a similar way. Furthermore, to clarify whether the DNA repair genes have different correlations between CNA and mRNA in tumours of distinct subtypes or stages, we performed the same analysis separately for each estrogen receptor (ER)-based subtype and for each tumour stage (Stage IV was not included as it is represented by only eight samples); the results (Supplementary Figures S1 and S2) are similar to those in Fig. 1. Thus for these DNA repair genes, CNA is likely to have a similar effect on gene expression across different types of breast tumours.

Table 2 displays the top ten repair genes whose differential expression is most likely due to their altered copy numbers (i.e., these ten genes have the highest correlations between CNA and mRNA). For example, the up-regulation of *POLR2K* can be largely ascribed to its copy number gain while the down-regulation of *POLR2C* is mainly due to its copy number loss. We consider that these relatively high correlations between inherent genetic changes and differential expression may have important implications for breast cancer therapy. For instance, recently studies showed that *CUL4A*, whose overexpression is associated with elevated drug sensitivity, is a promising biomarker for several cancers (including breast cancer)^24^,^25^, and here we revealed that *CUL4A* overexpression in breast cancer is mainly induced by its copy number gain. The protein encoded by *PARP1* is also a proposed drug target in breast cancer^26^, and here we showed that there is a relatively high correlation between its mRNA overexpression and DNA amplification (Table 2).

#### Contribution of DM to the DNA repair dysregulation

Compared to the correlations between CNA and mRNA, the correlations between DM and mRNA are generally weak, with a median value of about −0.25 (Fig. 2 and Supplementary Table S3). This is the case both for all differentially expressed repair genes, and for only those genes within each repair pathway (Fig. 2). Similar results were obtained for each ER-based subtype and each tumour stage (Supplementary Figures S3 and S4). We also found the DM-mRNA correlations are not significantly different between the up-regulated genes and down-regulated genes (p-value = 0.5, Wilcoxon rank-sum test). All these results suggest that DM is not a major factor for the differential expression of DNA repair genes in breast cancer, which is in line with a recent meta-analysis showing that cancer-specific methylation patterns usually have marginal effects on mRNA expression^27^.

A few DNA repair genes have modest correlations between DM and mRNA (Table 3). These genes are not enriched with downregulated repair genes (p-value = 1, Fisher’s exact test), indicating again that DM is not a major cause for the reduced repair gene expression in breast cancer. However, DM may have important effects on the underexpression of some genes listed in Table 3. For example, the transcriptional silencing of *WRN* by promoter hypermethylation is frequently observed in breast cancer^28^. This epigenetic inactivation can lead to increased chromosomal instability and hypersensitivity to DNA-damaging drugs, and thus has important implications for breast cancer therapy^28^,^29^.

Some DNA repair genes such as *BRCA1* and *PALB2* show relatively weak correlations between mRNA and DM (BRCA1, −0.32; PALB2, −0.40: Supplementary Table S3) and are thus not listed in Table 3; however, they may be regulated by promoter methylation in sub-populations of breast tumours. For example, *BRCA1* hypermethylation was observed in 13/143 (9.1%) sporadic breast tumours, most of which (9/13) also show diminished *BRCA1* expression^30^. Similarly, *PALB2* was found to be hypermethylated in 4/60 (6.7%) sporadic breast tumours and all four *PALB2* methylated tumours also exhibit low PALB2 expression^31^. Therefore, although DM is not likely to be a generally important factor affecting DNA repair gene expression in breast cancer, its role in DNA repair gene dysregulation in individual breast tumours needs to be investigated further.

### Estimation of the effects of TF transcriptional changes on the DNA repair dysregulation

#### TFs identified by our LASSO-based statistical framework and their contribution to the DNA repair dysregulation

To systematically search for TFs potentially involved in DNA repair dysregulation in breast cancer, we first downloaded a list of 1391 manually curated TFs that cover 85% to 94% of all the human TFs^32^. Next, for each of the 149 differentially expressed repair genes identified above, we built a linear regression model connecting CNA, DM and the transcriptional changes of the 1391 TFs to explain the observed repair gene dysregulation. Since the vast majority of the 1391 TFs are not associated with the dysregulation of a particular repair gene, we further developed a LASSO-based statistical framework to select relevant TFs for each repair gene (see Methods for detail).

In brief, the LASSO constraint^33^ enforces scarcity in a linear regression model (i.e., enforcing most of the small regression coefficients to be zero) and thus reduces the number of explanatory variables included in the model. To account for the effects of CNA and DM on gene expression, we imposed an additional constraint that the regression coefficients of CNA and DM will never be set to zero by LASSO. In other words, after taking into account the confounding effects from CNA and DM, we identified TFs whose transcriptional changes are associated with the aberrant expression of each repair gene. Through this approach, we selected 6 to 132 TFs (with a median value of 39) for each differentially expressed repair gene (Supplementary Table S4). Supplementary Table S5 summaries the Spearman correlations between the expression of a given repair gene and the expression of all of its selected TFs. Many of the selected TFs have established roles in DNA repair, and some of them are discussed in the next section.

To estimate the contributions of CNA, DM and TF-gene expression changes to repair gene dysregulation, we further constructed four alternative linear regression models for every differentially expressed repair gene. Each model uses the mRNA abundance of the same repair gene as the response variable, but comprises different explanatory variables as shown in Table 4. We compared the performance of the four models for the same repair gene via a subsampling-based process (see Methods for detail), and summarised the results across all the differentially expressed repair genes in terms of two measurements: Spearman correlation coefficient between predicted and observed mRNA abundance, and variance in the mRNA abundance explained by the model (coefficient of determination, R^2^).

As shown in Table 4, the model including only CNA performs better than the model with DM alone (average Spearman correlation coefficient 0.41 vs 0.25, and R^2^, 22% vs 0%), which is consistent with the result from Section 1 showing that CNA in general has a higher correlation with mRNA than DM. Table 4 also shows that, compared with using CNA alone, combining CNA and DM does not greatly improve the model performance (average Spearman correlation coefficient 0.44 vs 0.41, and R^2^, 24% vs 22%). By contrast, when the expression values of the selected TFs are added, the model performance becomes substantially improved (average Spearman correlation coefficient 0.85 vs 0.44, and R^2^, 73% vs 24%). Similar results (Supplementary Tables S6–S10) were obtained when the analysis described in this section (TF identification and model comparison) was conducted separately for each ER-based subtype and each tumour stage. All these results demonstrate that using the expression values of the identified TFs can substantially improve the model performance, which underscores the importance of these TFs in driving DNA repair dysregulation.

#### TFs that may be major drivers of DNA repair dysregulation

**Figure 3:**
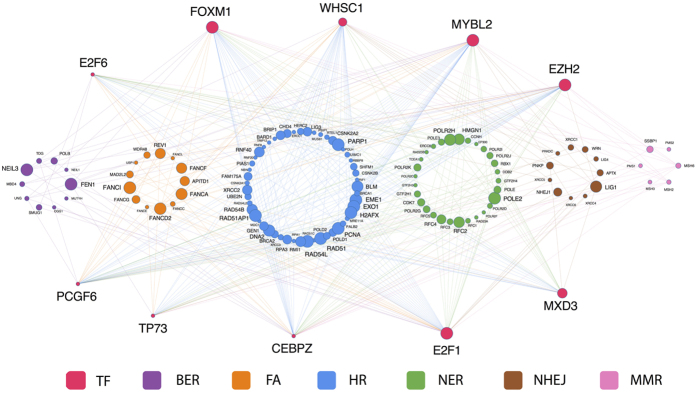
Ten TFs as potential master drivers of DNA repair dysregulation in breast cancer. TFs selected by the LASSO-based statistical framework were sorted by the number of their predicted DNA repair targets; only the top ten TFs and their targets are shown. The target repair genes are grouped according to pathway participation. Node size indicates level of differential expression.

Among the TFs identified by the LASSO-based statistical framework, some are predicted to target multiple genes within the same repair pathway, and therefore may be particularly important for the dysregulation of that pathway. Moreover, these TFs may also target genes that function in different repair pathways, and hence may be able to exert a global influence on the dysregulation of several repair pathways. With these thoughts in mind, we sorted the identified TFs according to the number of genes that they target. The top ten TFs and their pathway-specific targets are shown in Fig. 3. We consider these TFs as potential master drivers of DNA repair dysregulation in breast cancer.

Of these ten TFs, some have well-established roles in modulating DNA repair. The most prominent example is FOXM1, which is a master regulator of DNA damage response and a determinant of resistance to DNA-damaging agents^34^. Overexpression of the *FOXM1* gene is observed in many cancers^35^, including breast cancer^36^, and is thought to cause genomic instability^37^ and poor prognosis^38^,^39^. Another noted DNA repair regulator is E2F1, which coordinates the function of several vital cellular processes, including DNA repair, cell cycle checkpoint and apoptosis^40^,^41^,^42^. A recent study showed that, following treatment with histone deacetylase inhibitors (HDACs), a promising class of drug in prostate cancer, decreased recruitment of E2F1 results in downregulation of a few key DNA repair genes, leading to reduced DNA repair capacity and enhanced sensitivity to genotoxic agents^43^. Interestingly, most of these key repair genes, including *BRCA1*, *RAD51*, *RAD54L* and *BLM*, were also identified in this study as E2F1 targets in breast cancer.

Apart from TFs with well-established roles in DNA repair, the TFs shown in Fig. 3 also include those whose roles in DNA repair are less-well studied. For example, the protein p73 (also known as TP73), which belongs to the same family as the well-known tumour suppressor p53, was recently discovered to regulate DNA repair gene expression^44^. Moreover, it was previously reported that some tumour-derived P53 mutant proteins could negatively affect the function of the TP73 protein^45^, and we found that the expression of the p73 gene was significantly lower in p53 mutant tumours compared to tumours with wild-type p53 (p-value = 2.4e-07; Supplementary Figure S5). These results suggest that the involvement of TP73 in regulating repair genes might be more relevant to tumours with wild-type P53. As another example, MXD3, whose role in human DNA repair has not begun to be explored, was recently proposed to be involved in DNA repair in mice^46^. We hence propose that these TFs may serve as good candidates for identifying novel regulators of DNA repair and/or innovative drug targets for DNA repair-related breast cancer therapies.

#### TFs with ChIP-Seq profiles in ENCODE

In the LASSO-based statistical model, TFs were selected based on an association of the expression of the TF genes with the expression of a given repair gene. One issue associated with this process is that some of the TFs selected for a given repair gene may not directly regulate the repair gene, i.e. these TFs may function as upstream regulators of DNA repair, which do not directly bind and target a particular repair gene. We therefore sought other evidence that support the predicted TF-target relationships.

A major difficulty is that the genome-wide binding sites of most human TFs are currently unknown. For example, the Encyclopedia of DNA Elements (ENCODE) project, which aims to build a comprehensive list of functional elements in the human genome^47^, describes only 161 TFs (~10% of all known human TFs) that have ChIP-Seq data. These 161 TFs were profiled in 91 cell types, with each cell type having a few to dozens of TFs analysed (https://genome.ucsc.edu/encode/). In addition, for TFs whose binding sites have been measured by ChIP-Seq, how to define their direct target genes is still an open question^20^.

Here we searched the ENCODE database for TFs identified in this study and also with binding profiles measured by ChIP-Seq. As all ENCODE ChIP-Seq data were measured in cell lines, here we used the breast cancer cell line MCF-7 as a surrogate for the TCGA breast cancer samples analysed in this study. This cell line has been widely used in breast cancer research, and has more TFs measured by ChIP-Seq than do other breast cancer cell lines. Of the seven TFs measured in MCF-7, we found six (E2F1, MYC, TCF7L2, CTCF, GATA3, ZNF217) were identified in this study as potential DNA repair regulators. For each of these six TFs, we further examined how many of the predicted targets are potentially supported by the ChIP-Seq data. Specifically, we calculated the physical distances between TF binding sites and the transcription start sites (TSSs) of the target genes located on the same chromosome; we consider a direct TF-target relationship to exist if such a distance is ≤100 kb (the criterion was chosen according to^48^). As shown in Table 5, although the small sample size used in this analysis may lead to a biased result, we found in total 81% of the predicted DNA repair targets are potentially supported by the ChIP-Seq data; and in particular, of the 46 predicted E2F1 targets, 41 (89%) have supports from this ChIP-Seq analysis. This result suggests that most of the TFs identified in this study are likely to directly regulate their predicted DNA repair targets.

## Discussion

Prognostic and predictive biomarkers selected from high-throughput genomic data are of critical importance in cancer management^49^. Cancer-related dysregulation of DNA repair genes or pathways reflects altered DNA repair efficiency in tumours, and hence has been investigated intensively for biomarker discovery; to our knowledge, however, the genetic underpinnings of DNA repair dysregulation have not been systematically elucidated in any cancer type. Our results indicate that CNA and the transcriptional changes of TFs are major causes of DNA repair dysregulation in breast cancer, and some TFs may exert global impacts on the dysregulation of multiple DNA repair pathways. Our work thus provides novel biological insights into DNA repair dysregulation in breast cancer. These insights improve our understanding of the molecular basis of the DNA repair biomarkers identified thus far, and have potential to inform future biomarker discovery.

Access to multi-omics data for major cancer types has been greatly facilitated by large-scale projects such as TCGA in recent years. Accordingly, many methods for integrative multi-omics data analysis have emerged, aiming to help understand the interplay between different molecular levels, and/or provide improved power to identify important genomic factors^50^,^51^. Compared to other integrative methods, linear regression models have two distinct advantages for studying the altered transcriptional programs in cancer: 1) they regard the expression of a gene as a function of CNA, DM and TF activities etc., and thus provide a priming biological knowledge-based causal framework for data integration and gene expression modelling; and 2) unlike most integrative methods, which may encounter the “curse of dimensionality” when adding more data types into the analysis, linear regression models are quite flexible in this regard because even with a large number of potential explanatory variables, a parsimonious model can still be obtained through penalisation (e.g., by LASSO).

Studies that utilise linear regression models for multi-omics data analysis have been reported recently^52^,^53^,^54^,^55^. For instance, Li and colleagues^52^ fitted a linear model on the expression of each gene in acute myeloid leukaemia (AML) using gene-specific CNA, DM, TF binding signals and the counts of miRNA binding sites at the 3′-UTR as explanatory variables. In another study, Setty *et al.*^53^ modelled gene expression change in glioblastoma as a linear function of CNA, DM, the number of TF binding sites at the promoter region, and the number of miRNA binding sites at the 3′-UTR. These studies successfully identified a dozen TFs and miRNAs as key drivers of global transcriptional changes in AML and glioblastoma, respectively^52^,^53^.

However, these regression-based analyses also have certain limitations. Most importantly, while LASSO-enhanced linear regression models can achieve better prediction accuracy and interpretability by reducing the number of explanatory variables, the variable selection results can be significantly influenced by the choice of the initiating factor λ. As a common practice in the field, such as in the aforementioned study in AML^52^, the λ value is determined by running a cross-validation function only once, which may lead to an unstable result due to the random nature of the cross-validation process. In this study, we addressed this issue by developing a secondary feature selection procedure that ensures the robustness of the identified TFs (See Methods for detail).

Another problem is associated with insufficiency of explanatory variables. For example, the number of TFs covered by either of the two above-mentioned studies was quite limited. Specifically, Li and colleagues^52^ conducted TF identification from 97 TFs whose binding profiles were measured in K562, a cell line that by far has the highest number of TFs measured by ENCODE ChIP-Seq experiments; the TF binding information utilised by Setty *et al.*^53^, retrieved from the TRANSFAC database^56^, was available for only 152 TFs. Although TF-binding information from ChIP-Seq experiments or the TRANSFAC database can be more accurate, the vast majority of human TFs were nonetheless omitted from these studies. By contrast, in the current study we performed TF selection from a list of 1391 TFs, covering 85% to 94% of all human TFs. This high coverage enabled us to identify TFs potentially involved in DNA repair.

There are some remaining issues in this study that are mainly associated with lack of datasets. Firstly, our current model does not consider the impact of miRNAs on gene expression, i.e. key variables might be absent. In fact, we failed to establish an association between dysregulation of DNA repair gene and expression changes of miRNAs (data not shown). The reason might be that in comparison with TFs, miRNAs usually have much smaller effects on target gene expression^57^, and so given the large number of TFs in the model, miRNA-mediated downregulation was not recognised by our LASSO-based approach. Future studies employing other features of miRNA, and/or other genomic datasets may refine the current model. Secondly, due to a lack of other large breast cohorts measured at multiple molecular levels, we had to perform model training and testing on the same TCGA breast cancer dataset. This limitation is common to a number of recent studies^52^,^53^,^55^. Thirdly, the TFs selected in this study were mainly based on statistical analysis and thus may contain false positives. Although the results in Section 3.3 indicate that our result may enjoy high accuracy, we hope that in the future more experimentally based TF-binding profiles will be available to evaluate our results.

In summary, we performed a statistical analysis to dissect the roles of CNA, DM and the transcriptional changes of TFs in DNA repair dysregulation in breast cancer. Our results indicate that CNA and the transcriptional changes of TFs are major factors affecting the dysregulation of individual DNA repair genes, and that some TFs may be master drivers affecting several repair pathways. This work facilitates a mechanistic understanding of how the exquisite control of DNA repair regulation is pathologically altered in breast cancer, and thus may provide important implications for future DNA repair–based biomarker discovery. With the accumulation of the ever-increasing amount of genomic data and developments in integrative analysis methods, a complete understanding of DNA repair dysregulation in cancer will no longer beyond reach.

## Methods

### Data collection

The preprocessed genomic data generated by TCGA^15^, including the gene expression data for 113 normal breast tissues, and the gene expression and CNA data for 720 breast tumour samples were retrieved from the UCSC Cancer Genomics Browser (https://genome-cancer.ucsc.edu/). The preprocessed DM data for the same tumour set were retrieved from cBioportal (http://www.cbioportal.org/). The 720 breast tumours belong to different ER-based subtypes (511 samples are ER^+^, 153 are ER^-^ and 56 are with unknown ER status information) and are in different stages (112 samples in stage I, 394 in stage II, 183 in stage III, eight in stage IV and 23 with no stage information). Only samples annotated with ER status or stage information were included for the subtype- or stage-specific analysis. The gene expression data for both the normal tissues and tumour samples had been generated using the Illumina HiSeq 2000 RNA sequencing platform, and show the Expectation Maximisation (RSEM)-normalised and percentile-ranked gene-level transcription estimates. The CNA data had been produced using Affymetrix SNA 6.0 arrays, with germline copy-number variation filtered out. The CNA values we obtained are gene-level segmentation values where value 0 represents the diploid state of the chromosome. The DM profiles had been produced with the Illumina Infinium HumanMethylation450 platform. The preprocessed methylation values we obtained, known as beta values, are continuous variables between 0 and 1, representing the percentages of methylation at the gene promoter regions (defined as regions from 1.5 kb upstream to 0.5 kb downstream of transcription start sites).

The pre-processed ENCODE Chip-Seq data measured on the breast cancer cell line MCF-7 were downloaded from the UCSC genome browser (https://genome.ucsc.edu/encode/).

### Differential expression analysis

DNA repair genes differentially expressed in tumour versus normal breast tissues were identified using Limma^58^, with the criterion that false discovery rate (FDR) < 0.05 after Benjamini and Hochberg’s multiple-test adjustment^59^.

### Identification of TFs potentially involved in DNA repair dysregulation

To systematically search for TFs potentially involved in DNA-repair dysregulation in breast cancer, we performed TF selection from a list of 1391 manually curated human TFs, which was estimated to cover 85% to 94% of all human TFs^32^. Specifically, for each differentially expressed DNA repair gene identified in this study, we built a linear regression model connecting CNA, DM and the transcriptional changes of the 1391 TFs to explain the observed expression variance of the repair gene. For each repair gene *g*, we formulate the model as:


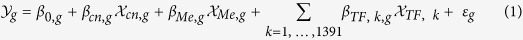


where 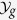, 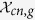 and 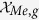 represent the abundances of mRNA, CNA, and DM of DNA repair gene *g*, respectively, while 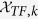 denotes the mRNA level of TF *k*. The regression coefficients 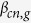 and 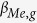 estimate the in *cis* contributions of CNA and DM to the expression changes of the repair gene *g*, while 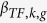 evaluates the influence of the transcriptional changes of TF *k* on the expression changes of repair gene *g*. The intercept is represented by 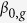 and error term by 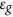.

We then applied the LASSO constraint^33^ through the R package *glmnet*^60^ to select a subset of the 1391 TFs whose transcriptional changes are significantly associated with the dysregulation of a given repair gene. To ensure that the effects of CNA and DM on gene expression are always taken into consideration, we imposed an additional constraint, also through the *glmnet* package, that the regression coefficients of CNA and DM are never set to zero by LASSO during this feature selection process.

**Figure 4:**
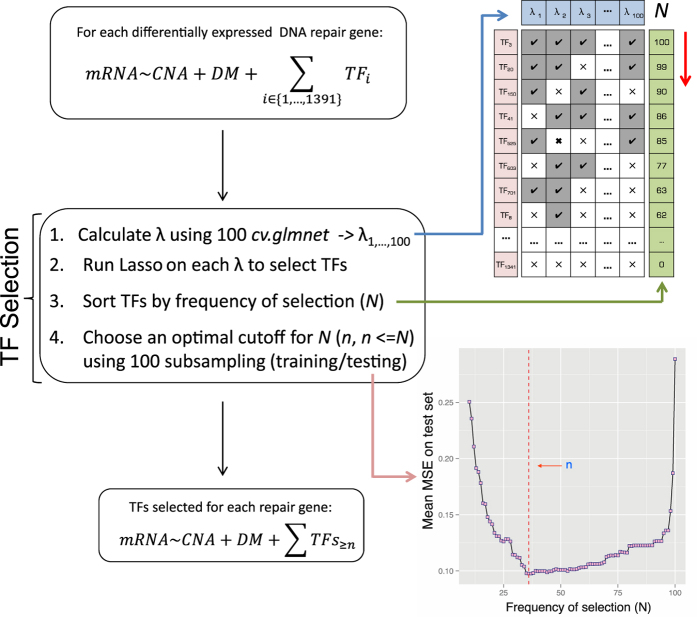
A LASSO-based statistical framework to select TFs potentially involved in DNA repair dysregulation.

In practice, a major drawback of LASSO is that its result can be heavily affected by an initiating parameter termed regularisation coefficient (λ), whose value needs to be specified for each analysis. This value is typically obtained using cross-validation; however, due to the randomness inherent to the cross-validation process, the estimated optimal λ value for the same analysis can differ across different cross-validation runs, resulting in unstable feature selection results. To overcome this, we developed a secondary feature-selection procedure with the assumption that TFs consistently selected with different λ values are likely to be truly associated with a given repair gene (Fig. 4).

Specifically, for each differentially expressed DNA repair gene, we generated 100 different λ values by running the *cv.glmnet* function 100 times, and then performed LASSO on each λ. This led to 100 different but overlapping sets of TFs selected for the same repair gene. Next, we calculated for each TF the frequency of being selected across the 100 LASSO runs. This frequency, denoted as 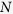
(1 ≤ *N* ≤ 100), is important as it indicates the selection stability for each TF. To determine an optimal cutoff for 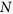, we further built different regression models, whose response and explanatory variables are similar to the one described above except that, instead of including all the 1391 TFs, only the TFs that had been selected more than 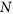
times were included. As different values of 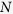 correspond to different sets of TFs, and in turn to different regression models, we reasoned that the optimal cutoff of 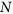 could be obtained by comparing the performances of all the possible models. For this purpose, in the following step we randomly divided the samples into a training set (2/3 of all samples) and a testing set (the remaining 1/3 of all samples) for model training and testing, respectively. We repeated this subsampling process 100 times, and each time the performance of each model on the testing set was recorded as the mean squared error (MSE). The value of 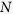 that gave the minimal averaged MSE across the 100 subsampling was considered the optimal cutoff, and the TFs whose selection frequency was above this cutoff were considered to be associated with a given DNA repair gene (Fig. 4).

### Four alternative linear regression models to estimate the contributions of possible mechanisms to DNA repair dysregulation

We constructed four alterative linear regression models for each differentially expressed DNA repair gene to estimate the contributions of CNA, DM and TF-gene expression changes to DNA repair gene dysregulation. Each model uses the mRNA abundance of the same DNA repair gene as response variable, but comprises different explanatory variables as follows:

1. DM only
2. CNA only
3. CNA + DM
4. CNA + DM + TFs

We compared the performances of these alternative models via a subsampling-based process. Specifically, we randomly selected two-thirds of the tumour samples to train each of the four models, and the remaining one-third of the samples was used for testing model performance. This process was repeated 100 times for each differentially expressed DNA repair gene, and the average performance of each model on the testing sets was recorded.

## Additional Information

**How to cite this article**: Liu, C. *et al.* Integrating Multi-omics Data to Dissect Mechanisms of DNA repair Dysregulation in Breast Cancer. *Sci. Rep.* **6**, 34000; doi: 10.1038/srep34000 (2016).

## Supplementary Material

Supplementary Information

Supplementary Table S1

Supplementary Table S2

Supplementary Table S3

Supplementary Table S4

Supplementary Table S5

## Figures and Tables

**Table 1:** Number of differentially expressed (DE) genes in each DNA repair pathway.

Pathway	Curated*	Present In TCGA	DE	Overexpressed	Underexpressed
HR	82	60	60	43	17
NER	66	48	48	36	12
BER	31	27	27	24	3
FA	31	23	23	19	4
NHEJ	25	22	22	13	9
MMR	24	20	20	15	5
Total†	195	169	149	106	43

^*^This column represents the number of manually curated genes in each repair pathway.

^†^Genes that appear in two or more pathways were counted only once.

**Table 2:** Top ten DNA repair genes sorted by their in *cis* correlations between CNA and mRNA expression.

Gene	Cor.	FDR	Pathway	Expression
POLR2K	0.78	6.59E-149	NER	Up
POLR2C	0.77	1.86E-140	NER	Down
CSNK2A2	0.77	9.37E-138	HR	Down
ERCC5	0.72	1.21E-116	NER	Down
RNF40	0.71	1.41E-111	HR	Up
CUL4A	0.70	2.64E-107	NER	Up
XRCC6	0.70	1.51E-105	NHEJ	Up
RAD54B	0.69	1.65E-100	HR	Up
TCEA1	0.66	9.51E-91	NER	Up
PARP1	0.60	6.68E-72	HR, NHEJ, BER	Up

**Table 3:** Top ten DNA repair genes sorted by their in *cis* correlations between DM and mRNA expression.

Gene	Cor.	p-value	Pathway	Expression
TCEA3	−0.55	2.16E-55	NER	Up
TCEA1	−0.47	8.09E-39	NER	Up
PARP3	−0.45	5.04E-36	NHEJ	Down
FANCA	−0.45	6.76E-35	FA	Up
RAD54B	−0.44	6.62E-34	HR	Up
PSIP1	−0.43	1.98E-32	HR	Down
WRN	−0.42	2.65E-31	NHEJ	Down
MUTYH	−0.41	4.85E-29	BER	Up
POLB	−0.41	6.00E-29	BER	Up
ERCC5	−0.40	6.83E-28	NER	Down

**Table 4:** Performance comparison of the four linear regression models.

	Spearman Correlation Coefficient (%)				Coefficient of Determination (R^2^) (%)			
	Min	Median	Mean	Max	Min	Median	Mean	Max
DM	−13	24	25	55	−237	3	0	24
CNA	−7	40	41	78	−14	20	22	61
CNA + DM	−6	43	44	78	−13	21	24	61
CNA + DM + TFs	64	86	85	97	34	74	73	91

Each model uses the mRNA abundance of the same DNA repair gene as the response variable, but comprises different explanatory variables listed in the first column. A negative R^2^ means that the linear model poorly fits the data.

**Table 5:** TFs with predicted DNA repair targets and ChIP-Seq profiles from ENCODE.

TF	No. of predicted targets	No. of predicted targets supported by ChIP-Seq data	Percentage
E2F1	46	41	89%
MYC	12	10	83%
TCF7L2	8	5	63%
CTCF	6	6	100%
GATA3	6	2	33%
ZNF217	2	1	50%
Total	80	65	81%
