# Supplementary material for: Integrating Multi-omics Data to Dissect Mechanisms of DNA repair Dysregulation in Breast Cancer
Source: Sci Rep. 2016 Sep 26;6:34000. doi: 10.1038/srep34000 (PMC5036051; doi:10.1038/srep34000)
Supplement: Supplementary Information [file srep34000-s1.pdf]

ONLINE DATA SUPPLEMENT

Integrating Multi-omics Data to Dissect Mechanisms of DNA repair Dysregulation in Breast Cancer

Chao Liu[1], Florian Rohart[2], Peter T. Simpson[3], Kum Kum Khanna[4], Mark A. Ragan[1*] and Kim-Anh Lê Cao[2*]

[1]Institute for Molecular Bioscience, The University of Queensland, St. Lucia, QLD 4067, Australia
[2]Diamantina Institute, The University of Queensland, Woolloongabba, QLD 4102, Australia
[3]UQ Centre for Clinical Research and School of Medicine, The University of Queensland, Herston, QLD 4101, Australia
[4]QIMR-Berghofer Medical Research Institute, Herston, Brisbane, QLD 4006, Australia

[*] Joint corresponding authors:

Kim-Anh Lê Cao, Diamantina Institute, The University of Queensland, 37 Kent Street, Woolloongabba, QLD 4102, Australia. Tel: +61 7 3443 7069; Fax: +61 7 3443 6966; Email: k.lecao@uq.edu.au

Mark A. Ragan, Institute for Molecular Bioscience, The University of Queensland, 306 Carmody Rd, St. Lucia, QLD 4067, Australia. Tel.: +61 7 3346 2616; Fax: +61 7 3346 2101; E-mail: m.ragan@uq.edu.au

a

b

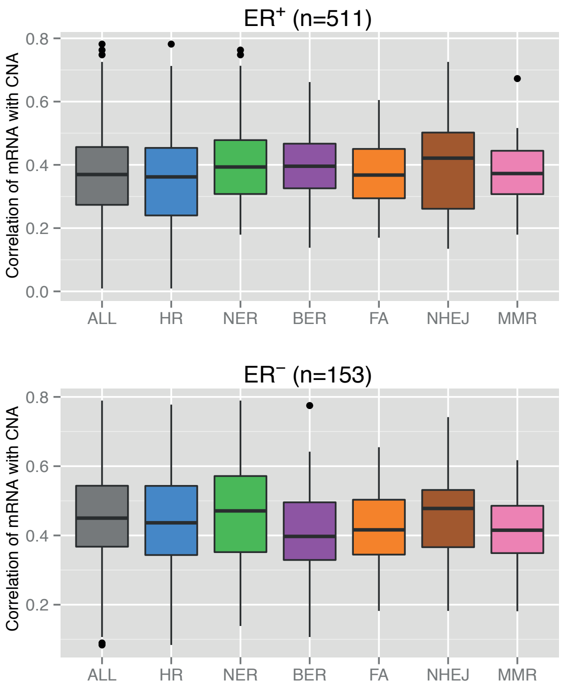

Fig. S1. Related to Fig. 1. The effects of CNA on DNA repair gene expression in different estrogen receptor (ER)-based breast cancer subtypes. Distributions of the in *cis* Spearman correlations between CNA and mRNA expression, summarised for all differentially expressed DNA repair genes, or only genes from each repair pathway.

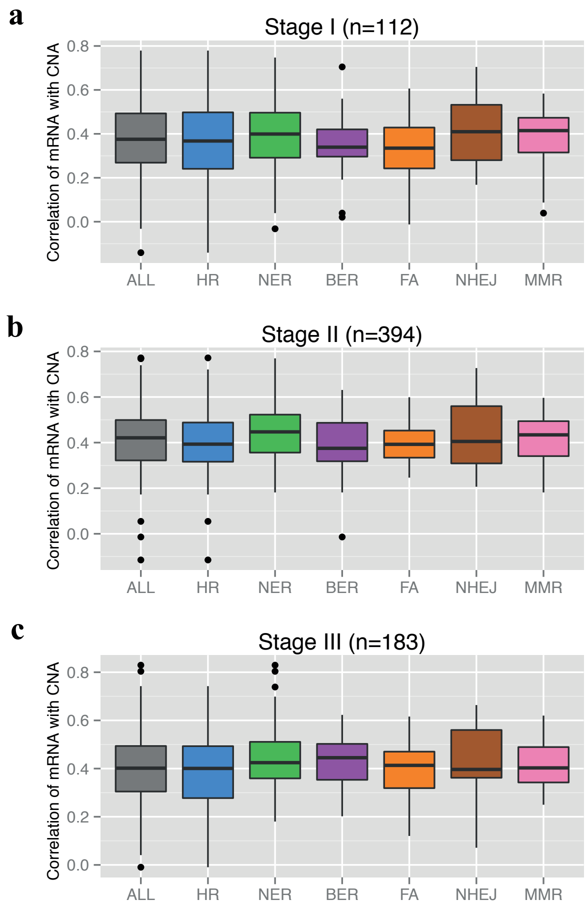

Fig. S2. Related to Fig. 1. The effects of CNA on DNA repair gene expression in different breast tumour stages. Distributions of the in *cis* Spearman correlations between CNA and mRNA expression, summarised for all differentially expressed DNA repair genes, or only genes from each repair pathway. Stage IV was not included as it is represented by only eight samples.

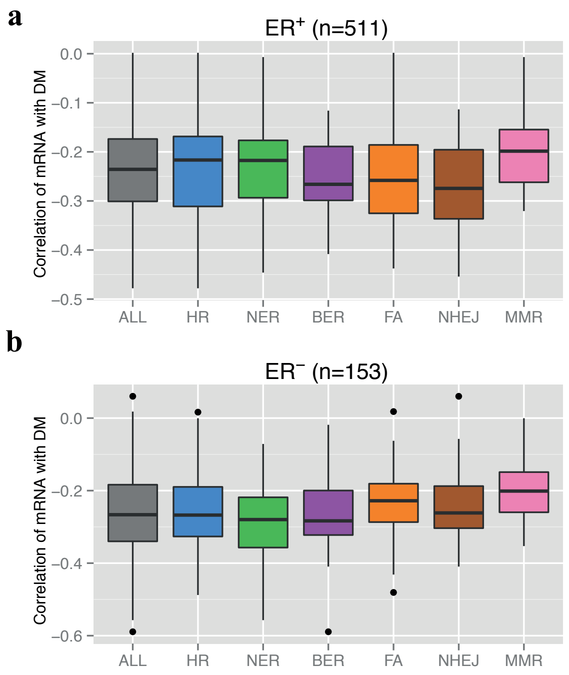

Fig. S3. Related to Fig. 2. The effects of DNA methylation (DM) on DNA repair gene expression in different estrogen receptor (ER)-based breast cancer subtypes. Distributions of the in *cis* Spearman correlations between DM and mRNA expression, summarised for all differentially expressed DNA repair genes, or only genes from each repair pathway.

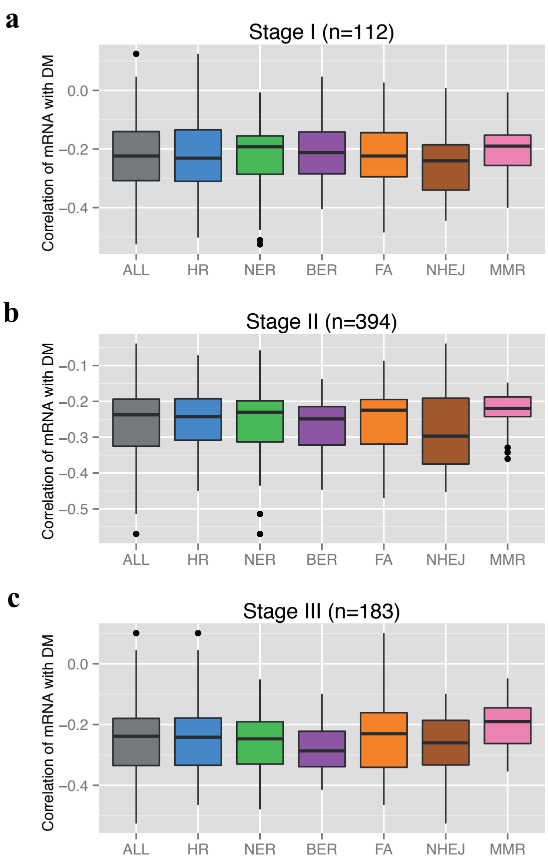

Fig. S4. Related to Fig. 2. The effects of DNA methylation (DM) on DNA repair gene expression in different breast tumour stages. Distributions of the in *cis* Spearman correlations between DM and mRNA expression, summarised for all differentially expressed DNA repair genes, or only genes from each repair pathway. Stage IV was not included as it is represented by only eight samples.

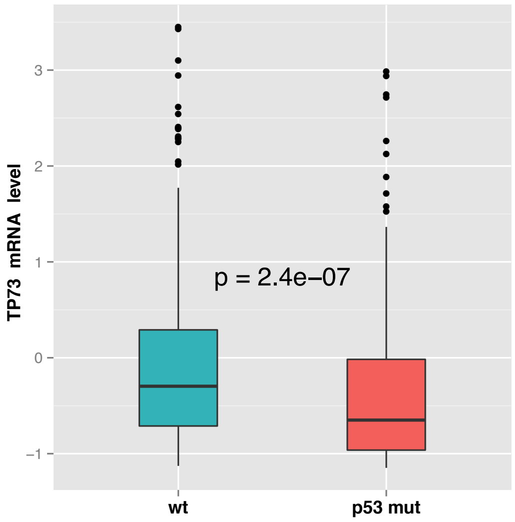

Fig. S5. The *TP73* gene expression in *p53* mutant versus *p53* wild-type breast tumours. There are 190 samples with *p53* mutant and 445 with *p53* wild-type. Samples not annotated with *p53* mutation status were excluded from the analysis. The p-value was obtained using a Wilcoxon rank-sum test.

Supplementary Table S6. Related to Table 4. Performance comparison of the four linear regression models for ER^+ breast tumours. Each model uses the mRNA abundance of the same DNA repair gene as the response variable, but comprises different explanatory variables as listed below. A negative R^2 means that the linear model poorly fits the data.

	Spearman Correlation Coefficient (%)				Coefficient of Determination (R^2) (%)			
	Min	Median	Mean	Max	Min	Median	Mean	Max
DM	-19	23	23	45	-276	2	-3	19
CNA	-2	37	37	78	-51	18	20	63
CNA + DM	-5	40	40	79	-50	19	21	63
CNA + DM + TFs	59	87	85	97	35	75	73	92

Supplementary Table S7. Related to Table 4. Performance comparison of the four linear regression models for ER^- breast tumours. Each model uses the mRNA abundance of the same DNA repair gene as the response variable, but comprises different explanatory variables as listed below. A negative R^2 means that the linear model poorly fits the data.

	Spearman Correlation Coefficient (%)				Coefficient of Determination (R^2) (%)			
	Min	Median	Mean	Max	Min	Median	Mean	Max
DM	-10	26	24	58	-41	0	0	24
CNA	-5	45	45	78	-35	16	18	67
CNA + DM	-7	46	46	80	-36	17	18	71
CNA + DM + TFs	52	83	82	94	20	70	67	91

Supplementary Table S8. Related to Table 4. Performance comparison of the four linear regression models for stage I breast tumours. Each model uses the mRNA abundance of the same DNA repair gene as the response variable, but comprises different explanatory variables as listed below. A negative R^2 means that the linear model poorly fits the data.

	Spearman Correlation Coefficient (%)				Coefficient of Determination (R^2) (%)			
	Min	Median	Mean	Max	Min	Median	Mean	Max
DM	-15	20	19	50	-1227	-6	-18	15
CNA	-8	37	37	77	-20	10	13	55
CNA + DM	-3	39	39	77	-104	9	11	54
CNA + DM + TFs	50	84	82	96	-5	71	70	92

Supplementary Table S9. Related to Table 4. Performance comparison of the four linear regression models for stage II breast tumours. Each model uses the mRNA abundance of the same DNA repair gene as the response variable, but comprises different explanatory variables as listed below. A negative R^2 means that the linear model poorly fits the data.

	Spearman Correlation Coefficient (%)				Coefficient of Determination (R^2) (%)			
	Min	Median	Mean	Max	Min	Median	Mean	Max
DM	-6	24	25	57	-437	2	-4	25
CNA	-8	42	41	77	-58	18	21	58
CNA + DM	-6	43	44	79	-145	20	21	61
CNA + DM + TFs	53	85	84	96	-57	73	71	89

Supplementary Table S10. Related to Table 4. Performance comparison of the four linear regression models for stage III breast tumours. Each model uses the mRNA abundance of the same DNA repair gene as the response variable, but comprises different explanatory variables as listed below. A negative R^2 means that the linear model poorly fits the data.

	Spearman Correlation Coefficient (%)				Coefficient of Determination (R^2) (%)			
	**Min**	**Median**	**Mean**	**Max**	**Min**	**Median**	**Mean**	**Max**
**DM**	-18	23	22	51	-641	-1	-5	22
**CNA**	-3	40	40	82	-48	15	17	64
**CNA + DM**	2	42	42	81	-29	16	17	63
**CNA + DM + TFs**	46	84	82	95	-6	72	69	91
